# The effect of maternal concerns about childbirth and postpartum period on obsessive and compulsive behaviors related to baby care

**DOI:** 10.7705/biomedica.7146

**Published:** 2024-08-29

**Authors:** Dilek Menekşe, Özge Karakaya Suzan, Nursan Çinar

**Affiliations:** 1 Department of Peadiatric Nursing, Faculty of Health Sciences, Sakarya University, Sakarya, Türkiye Sakarya University Sakarya University Sakarya Sakarya

**Keywords:** Labor, obstetric, postpartum period, mothers, trabajo de parto, periodo posparto, madres

## Abstract

**Introduction.:**

Postpartum anxiety after childbirth is a common condition among pregnant women due to reasons such as the uncertainty of experiencing pregnancy and childbirth for the first time, or previous negative experiences. Fear of childbirth can affect the mother’s baby care process.

**Objective.:**

This study was conducted analytically with a single-subject design to determine the effects of maternal concerns about childbirth and the postpartum period on obsessive and compulsive behaviors related to baby care.

**Materials and methods.:**

The study was conducted with 260 mothers. Data were collected using a descriptive information form, and the scales ‘Fear of Childbirth and Postpartum Period’, and ‘Obsessive and Compulsive Behaviors of Mothers in the Postpartum Period Related to Baby Care’. The data were analyzed using the SPSS™ software to calculate percentages, mean values, t tests, ANOVA, Pearson’s correlation, and simple linear regression analysis.

**Results.:**

A statistically significant and positive correlation was found between participant scores of the ‘Fear of Childbirth and Postpartum Period’ and the ‘Obsessive and Compulsive Behaviors of Mothers in the Postpartum Period Related to Baby Care’ scales (p < 0.01). The regression model showed that 18.0% of the total variance in the obsessive and compulsive behaviors of mothers in the postpartum was explained by the fear of childbirth and the postpartum period (corrected R^2^ = 0.180).

**Conclusions.:**

Fear of childbirth and the postpartum period were moderate. However, as the fear of women regarding childbirth and the postpartum period increased, their postpartum obsessive and compulsive behaviors about baby care also increased.

## Resumen

**Introducción.:**

La ansiedad del parto y el posparto es una condición común entre las mujeres embarazadas por la incertidumbre de vivir el embarazo y el parto por primera vez o por previas experiencias negativas. El miedo al parto puede afectar el proceso del cuidado del bebé de la madre.

**Objetivo.:**

Determinar el efecto de las preocupaciones maternas sobre el parto y el puerperio y su efecto sobre las conductas obsesivas y compulsivas relacionadas con el cuidado del bebé.

**Materiales y métodos.:**

El estudio se realizó con 260 madres. Los datos fueron recolectados en el formulario de información descriptiva y se usaron la “Escala de miedo al parto y al período posparto” y la de “Comportamientos obsesivos y compulsivos de las madres en el período posparto relacionados con el cuidado del bebé”. Los datos fueron evaluados mediante el software SPSS™ mediante el cálculo de porcentajes, promedio, prueba t, ANOVA, correlación de Pearson y análisis de regresión múltiple.

**Resultados.:**

Se encontró una correlación positiva y estadísticamente significativa entre la “Escala de miedo al parto y del período posparto” y la de “Comportamientos obsesivos y compulsivos de las madres en el período posparto relacionadas con el cuidado del bebé” (p < 0.01). En el modelo creado por análisis de regresión se observó que el 18,0% del cambio en la escala de comportamientos obsesivos-compulsivos estaba explicado por el miedo al parto y al puerperio (R^2^corregido = 0,180).

**Conclusiones.:**

En el estudio se determinó que el miedo al parto y al puerperio era moderado. Sin embargo, a medida que aumentaba el puntaje de miedo al parto y al período posparto, también aumentaban los comportamientos obsesivos y compulsivos de las madres en el puerperio relacionados con el cuidado del bebé.

Postpartum anxiety following childbirth is a common condition among pregnant women due to reasons such as the uncertainty of experiencing pregnancy and childbirth for the first time and previous negative experiences ^1^^,^^2^. The severe experience of this fear (tokophobia) negatively affects women’s activities of daily living, pregnancy, and childbirth period, as well as their approach to the baby after birth and the baby care period ^3^. In this context, it is undeniable that the processes of pregnancy, childbirth, and postpartum are interconnected.

Obsessive-compulsive disorder, which is among prominent anxiety disorders, is characterized by the presence of obsessions and/or compulsions ^4^. Pregnancy and childbirth have long been defined as events of onset or exacerbation in retrospective studies of the obsessive-compulsive disorder ^5^^,^^6^. In a recent meta-analysis study conducted by high-quality methodology, the prevalence of postpartum obsessive-compulsive disorder was between 1.7 and 2.2% overall ^7^.

Studies in recent years have focused on these symptoms’ effects on mothers and their babies ^5^^,^^8^^-^^10^. In the phenomenology of perinatal obsessive-compulsive disorder symptomatology, while contamination fears appear to be more prominent during pregnancy, having intrusive thoughts about the avoidance of intentionally harming the baby seems to be a common postpartum attitude ^5^.

Obsessions are a frequent feature of other mood and anxiety disorders arising in the perinatal period. At least 65% of new parents reported experiencing intrusive thoughts, though for many, these were transient and did not affect their functioning ^11^. Behaviors in obsessive-compulsive disorders become worse by the condition -potential negative- effects on infant development, mother-infant interaction, and with the other parent ^5^^,^^11^.

In postpartum obsessive-compulsive disorder cases, contagion and other obsessive themes are generally related to the baby, may cause high stress and reduce the person’s contact with the baby ^5^^,^^12^. Untreated postpartum anxiety has been associated with adverse effects such as unhealthy motherinfant communication, impaired child development, and low maternal self-confidence in infant care ^5^^,^^13^. Additionally, the uncertainties of the COVID-19 pandemic have increased the prevalence of these potential issues.

Considering this information, this study was conducted to determine the effects of maternal concerns about childbirth and the postpartum period on obsessive and compulsive behaviors related to baby care during the active phase of the COVID-19 pandemic.

This study answered the following questions:

1. Is there a relationship between maternal concerns about childbirth and the postpartum period and obsessive and compulsive behaviors related to baby care?
2. What are the factors affecting mothers’ concerns about childbirth and the postpartum period and their obsessive and compulsive behaviors related to baby care?

## Materials and methods

### Design and setting

This prospective analytical study was conducted with pregnant women in a hospital in the western part of Türkiye between June 2020 and July 2021.

### Participants

The studied population consisted of pregnant women who attended the obstetrics and gynecology outpatient clinics of a research and training hospital. The sample consisted of 273 mothers who met the inclusion criteria, agreed to participate after being informed by the researchers, and filled out the data collection forms completely. The minimum sample size required for the study was 134 participants with a significance level of 0.05, a power of 95%, and a medium size effect, using the G*Power 3.1.9 statistical analysis software.

The inclusion criteria were as follows:

- Being a pregnant woman over 19 years old.
- Not having a risky pregnancy (e.g., preeclampsia, eclampsia, threat of preterm labor).
- Consent to fill in the “Scale of Obsessive and Compulsive Behaviors of Mothers in the Postpartum Period Related to Baby Care” by phone after delivery.
- Not having a prior diagnosis of obsessive-compulsive disorder.
- Being in the third trimester of pregnancy.
- Agreeing to participate in the study.

The exclusion criteria were:

- Having preterm babies (n = 3).
- Having newborns hospitalized in the intensive care unit (n = 3).
- Being unreachable by phone (n = 7).

**Figure 1:**
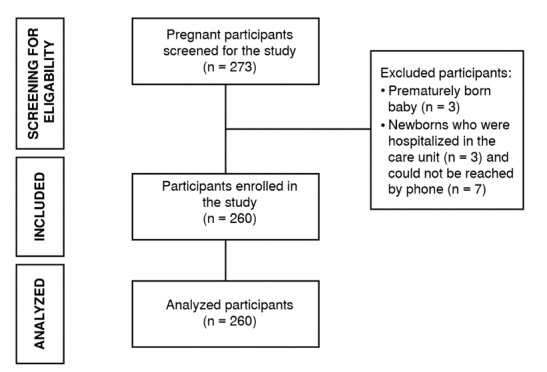
Study participants flow diagram

As a result of screening for the inclusion and exclusion criteria, 260 mothers were included in the study (figure 1).

### Variables

*Independent variables:* Fear of childbirth and the postpartum period

*Dependent variables:* Obsessive and compulsive behaviors of mothers in the postpartum period regarding baby care

### Data collection tools

The descriptive information form consisted of 17 questions to determine participants’ sociodemographic and obstetric characteristics. Sociodemographic questions were related to age, education and employment status, and family type; and obstetric questions concerned week of gestation, number of pregnancies, intended pregnancy status, and having problems during pregnancy.

The Fear of Childbirth and Postpartum Period Scale (FCPPS) was developed by Kitapçioglu et al. in 2008. It consists of 10 dimensions (61 items) designed to identify the concerns experienced by women in the childbirth and postpartum periods, and it has a five-point Likert-type scale where each item has a score in the range of 1-5 ^14^. There are no inversely scored items. The scores obtained after standardization ranged from 1 to 10 and were categorized as follows: 0.00-2.00: very low; 2.01-4.00: low; 4.016.00: medium, 6.01-8.00: high, and 8.01-10.00: very high. The validation analyses of the original scale showed a Kaiser-Meyer-Olkin coefficient of 0.868, a Bartlett’s test statistic of 8,906.8, and a 2,211 degrees of freedom value (p = 0.00001). We found 15 factors in the principal component analysis of the scale. The variance explained by the factors varied between 1.90 and 11.10%, and all factors collectively explained 71.86% of the total variance ^14^. In the original study of FCPPS, the Cronbach’s alpha coefficient of the overall scale was reported as 0.95. The Cronbach’s alpha coefficient of the scale in the sample of this study was 0.97.

The scale for Obsessive and Compulsive Behaviors of Mothers in the Postpartum Period Regarding Baby Care (OCB-PPBC) was developed by Õzdemir et al. in 2019 to determine, as its name suggests, the obsessive and compulsive behaviors of mothers related to baby care in the postpartum period and whose validity and reliability was checked, and it consists of nine items ^15^. Each item of the five-point Likert-type scale has response options scored between 1 and 5. The response options of the items are as follows: 1: Doesn’t describe me at all, 2: Describes me very little, 3: Describes me a little, 4: Describes me mostly, and 5: Describes me a lot. OCB-PPBC results are evaluated based on the total score. The lowest and highest scores that can be obtained from the scale are 9 and 45. A high score indicates that the woman has an increased obsessive and compulsive behavior related to baby care. In the validation analyses of the scale, the Kaiser-Meyer-Olkin test result was 0.809, and the Bartlett test of sphericity statistic was found significant (p < 0.01). As a result of exploratory factor analysis, the nine items were combined under a single factor. These items explained 34% of the total variance in the scale scores ^15^. The Cronbach’s alpha internal consistency coefficient of the scale was reported as 0.75, while this coefficient was found to be 0.85 in this study.

### Data collection

The purpose of the study was explained to pregnant women who met the inclusion criteria and attended the obstetrics and gynecology outpatient clinic of the hospital. Verbal and written consents were obtained from the pregnant

women after informing them about data confidentiality. Pregnant women who did not want to participate in the study were excluded. Each participant filled out the descriptive information for’ and FCPPS. Participant’s contact information was obtained to call them the first month after delivery. When the researcher and the participant were available, they filled out the OCB-PPBC in approximately five minutes.

### Ethical aspects of the research

The Health Ethics Committee of Sakarya University, Sakarya, Türkiye approved the study (Date: 27.01.2020, Issue:1522473/050.01.04/15). Institutional permission was obtained from the hospital where the study was conducted. This work was conducted in accordance with the principles of the Declaration of Helsinki. Before starting the study, researchers informed the eligible pregnant women about the study’s purpose and data privacy. Verbal and written consents were obtained from those who agreed to participate.

### Statistical analyses

The data collected in the study were analyzed using the SPSS™ (Statistical Package for the Social Sciences) for Windows 22.0 bundled software. We calculated frequency distributions for the categorical variables and descriptive statistics (mean, standard deviation, minimum, and maximum values) for the numerical variables. The independent-sample t test was used whether the values of the quantitative variables differed significantly from each other between two groups, and one-way analysis of variance (ANOVA) was used to test the differences among more than two groups. The Bonferroni test was used as a post hoc test to identify the sources of significant differences observed in the ANOVA analysis. Additionally, Pearson’s correlation and simple linear regression analysis were used to determine the direction and strength of the relationships between the scale and subscale scores of the participants. Cronbach’s alpha was used to test the reliability of the scales for data collection. The threshold for statistical significance was p < 0.05.

## Results

The distribution of the participants according to their sociodemographic characteristics is in table 1. The mean age of the participants was 27.96 ± 5.11 (minimum = 19; maximum = 40). Statistically significant differences were not found between the mean of FCPPS and OCB-PPBC scores of the participants based on their education level, family type, and economic status (p > 0.05). Also, no statistically significant difference in the mean of FCPPS was found in terms of participant’s employment status, but a significant one was found in the mean of the FCPPS scores based on their OCB-PPBC scores. Not working participants had a significantly higher OCB-PPBC mean score than those working (p < 0.05).

**Table 1:** Relationship between the sociodemographic characteristics and the mean of FCPPS and OCB-PPBC total scores of the participants (N = 260).

		n (%)	FCPPS	OCB-PPBC
			total	total
			**X̅ ± SS**	**X̅ ± SS**
Education level				
	Primary school	100 (38.5)	4.91 ± 1.46	25.26 ± 9.38
	High school	88 (33.8)	5.23 ± 1.38	23.70 ± 7.93
	University	72 (27.7)	5.07 ± 1.54	22.86 ± 8.52
	f /p		1.127 / 0.325	1.718 / 0.181
Employment status				
	Employed	46 (17.7)	4.99 ± 1.50	21.72 ± 8.32
	Unemployed	214 (82.3)	5.08 ± 1.45	24.57 ± 8.72
	t/p		-0.383 / 0.702	-2.033 / 0.043*
Economic status				
	Income is less than expenses	50 (19.2)	5.02 ± 1.66	25.90 ± 9.62
	Income is equivalent to expenses	193 (74.2)	5.10 ± 1.43	23.65 ± 8.52
	Income is more than expenses	17 (6.5)	4.69 ± 1.11	23.41 ± 7.62
	F/p		0.642 / 0.527	1.381 / 0.253
Family type				
	Nuclear family	219 (84.2)	5.13 ± 1.48	24.38 ± 8.61
	Extended family	41 (15.8)	4.70 ± 1.31	22.39 ± 9.06
	t/p		1.740 / 0.083	1.349 / 0.179
Smoking				
	Yes	36 (13.8)	5.06 ± 1.55	25.39 ± 7.98
	No	224 (86.2)	5.06 ± 1.45	23.86 ± 8.81
	t/p		-0.013 / 0.990	0.981 / 0.328
Chronic disease condition				
	Yes	27 (10.4)	5.30 ± 1.70	22.19 ± 8.01
	No	233 (89.6)	5.03 ± 1.43	24.29 ± 8.76
	t/p		0.885 / 0.377	-1.190 / 0.235

FCPPS: Fear of Childbirth and Postpartum Period Scale; OCB-PPBC: Obsessive and Compulsive Behaviors-Postpartum Period Baby Care; SS: Sum of squares

F: One Way Anova; t: Independent sample t test

*p < 0.05

The mean gestational week was 36.14 ± 3.32 (minimum = 24; maximum = 41). The participant’s characteristics distribution regarding their pregnancies and babies is in table 2. We found statistically significant relationships between the mean of the FCPPS and the OCB-PPBC scores and whether they were primiparous or multiparous (p < 0.05). Participants who had their first baby had significantly higher anxiety levels about childbirth and postpartum periods and significantly higher obsessive-compulsive behaviors related to baby care. No statistically significant difference was found in the mean of the FCPPS or the OCB-PPBC scores based on whether they had an intended pregnancy, experienced any health problems, had received education about the childbirth process and baby care, or had problems in their previous pregnancy, childbirth, and baby (p > 0.05).

**Table 2:** Relationship between participants' characteristics regarding pregnancy and baby and FCPPS and OCB-PPBC total mean scores (N = 260)

Features		n (%)	FCPPS	OCB-PPBC
			total	total
			**X̅ ± SS**	**X̅ ± SS**
Pregnancy				
	Primiparous	87 (33.5)	5.59 ± 1.32	25.80 ± 8.36
	Multiparous	173 (66.5)	4.78 ± 1.45	23.19 ± 8.5
	t/p		4.364 / 0.000**	2.300 / 0.022*
Planned pregnancy				
	Yes	181(69.6)	5.16 ± 1.40	23.43 ± 8.79
	No	79 (30.4)	4.84 ± 1.58	25.53 ± 8.37
	t/p		1.612 / 0.108	-1.799 / 0.073
Having any health problems during pregnancy				
	Yes	39 (15.0)	5.46 ± 1.38	25.13 ± 8.39
	No	221(85.0)	4.99 ± 1.47	23.88 ± 8.76
	t/p		1.849 / 0.066	0.824 / 0.411
Receiving education about the birth process				
	Yes	24 (9.2)	4.82 ± 1.70	21.58 ± 7.35
	No	236 (90.8)	5.08 ± 1.43	24.32 ± 8.80
	t/p		-0.843 / 0.400	-1.473 / 0.142
Receiving education about baby care				
	Yes	40 (15.4)	5.11 ± 1.65	23.33 ± 7.87
	No	220 (84.6)	5.05 ± 1.43	24.20 ± 8.85
	t/p		0.231 / 0.817	-0.587 / 0.557
Having problems in previous pregnancies				
	Yes	36 (20.7)	4.45 ± 1.38	22.08 ± 8,56
	No	138 (79.3)	4.84 ± 1.47	23.48 ± 8,77
	t/p		-1.408 / 0.161	-0.858 / 0.392
Having problems in previous births				
	Yes	27 (15.5)	4.88 ± .99	22.29 ± 7.63
	No	147 (84.5)	4.74 ± 1.53	23.36 ± 8.92
	t/p		0.604 / 0.548	-0.648 / 0.521
Having problems with previous babies				
	Yes	26 (14.9)	4.34 ± 1.22	22.61 ± 6.80
	No	148 (85.1)	4.83 ± 1.49	23.29 ± 9.03
	t/p		-1.574 / 0.117	-0.447 / 0.657
Delivery type			
	Vaginal	118 (45.4)	5.06 ± 1.44	24.76 ± 8.74
	Cesarean	142 (54.6)	5.06 ± 1.48	23.49 ± 8.65
	t/p		-0.051 / 0.960	1.173 / 0.242
Presence of a condition requiring hospitalization of the baby^¥^			
	Yes	27 (10.4)	-	23.26 ± 7.45
	No	233 (89.6)	-	24.16 ± 8.84
	t/p		-	-0.510 / 0.610

OCB-PPBC: Obsessive and Compulsive Behaviors-Postpartum Period Baby Care; FCPPS: Fear of Childbirth and Postpartum Period Scale; t: Independent sample t test

* p < 0.05

** p < 0.01

^¥^ Specified characteristics compared only to OCB-PPBC.

No statistically significant relationship was found between the mean of the OCB-PPBC scores and the delivery mode or the presence of a condition requiring the baby’s hospitalization (p > 0.05). The participants had an OCB- PPBC mean score of 24.07 ± 8.70 and an FCPPS mean score of 5.06 ± 1.46. These data indicated moderate levels of fear of childbirth and the postpartum period in general. Statistically significant and negative correlations were found between the OCB-PPBC scores and participant’s age (r = -0.129, p < 0.05), concerns about delivery (r = -0.156, p < 0.05), and concerns about inadequate baby care after childbirth (r = -0.155, p < 0.05) (table 3). Table 3 shows a statistically significant and positive correlation between the FCPPS and the OCB-PPBC scores (r = 0.429, p < 0.01).

**Table 3:** Relationship between age and scales

Variables		1	2	3	4	5	6	7	8	9	10	11	12	13
1.	Age	-												
2.	OCB-PPBC¥	-0.129*	-											
3.	Concerns about the baby	-0.120	0.359**	-										
4.	Concerns about labor	-0.156*	0.359**	0.728**	-									
5.	Concerns about postpartum breastfeeding	-0.078	0.368**	0.526**	0.676**	-								
6.	Concerns about inadequate baby care after birth	-0.155*	0.360**	0.550**	0.597**	0.544**	-							
7.	Concerns about postpartum social life	0.015	0.256**	0.345**	0.386**	0.371**	0.622**	-						
8.	Concerns about baby and women health after childbirth	-0.065	0.368**	0.561**	0.627**	0.606**	0.708**	0.696**	-					
9.	Concerns of not getting support from spouse after delivery	-0.076	0.255**	0.401**	0.456**	0.382**	0.604**	0.615**	0.658**	-				
10.	Concerns before labor	-0.077	0.376**	0.548**	0.655**	0.573**	0.571**	0.410**	0.614**	0.562**	-			
11.	Concerns about health personnel’s behavior during delivery	-0.108	0.269**	0.492**	0.651**	0.595**	0.457**	0.348**	0.547**	0.377**	0.569**	-		
12.	Concerns about cesarean section	-0.076	0.223**	0.404**	0.476**	0.417**	0.403**	0.361**	0.494**	0.428**	0.449**	0.433**	-	
13.	FCPPS^§^	-0.119	0.429**	0.744**	0.840**	0.773**	0.801**	0.673**	0.856**	0.709**	0.800**	0.744**	0.557**	-

OCB-PPBC: Obsessive and Compulsive Behaviors-Postpartum Period Baby Care; FCPPS: Fear of Childbirth and Postpartum Period Scale

* p < 0.05

** p < 0.01

According to other correlation results, there were statistically significant and positive correlations between the participant’s obsessive and compulsive behaviors related to baby care and their concerns about the baby (r = 0.359, p < 0.01), labor (r = 0.359, p < 0.01), postpartum breastfeeding (r = 0.368, p < 0.01), inadequate postnatal care (r = 0.360, p < 0.01), postpartum social life (r = 0.256, p < 0.01), baby and maternal health after childbirth (r = 0.368, p < 0.01), fear of not getting support from their partners after delivery (r = 0.255, p < 0.01), the moments before labor (r = 0.376, p < 0.01), the behaviors of health personnel during delivery (r = 0.269, p < 0.01), having a cesarean section delivery (r = 0.223, p < 0.01).

According to the F value significance in table 4, the established regression model was statistically significant (F = 58.035; p < 0.05). Based on the t value and the significance level of the β coefficients of the independent variables, the FCPPS scores had a statistically significant effect on the mother’s obsessive and compulsive behaviors related to baby care in the postpartum period (β = 0.429; p < 0.05). Eighteen percent of the total variance in the obsessive and compulsive behaviors related to baby care in the postpartum could be explained by participant’s fears of childbirth and the postpartum period (corrected R^2^ = 0.180).

**Table 4:** Participants’ simple linear regression analysis results to explain the effect of the fear of childbirth and postpartum period scale on obsessive-compulsive behaviors of mothers related to baby care in the postpartum period.

Dependent variable	Independent variable	β	t	p	Beta	F	Model (p)	Adjusted R^2^
OCB-PPBC	Constant FCPPS	11.147	1.765	0.000	0.429	58.035	0.000	0.180
		2.554	0.335	0.000**				

B: Unstandardized β coefficient; Beta: Standardized β, F: model statistics, R^2^: Correlation coefficient squared

OCB-PPBC: Obsessive and Compulsive Behaviors-Postpartum Period Baby Care; FCPPS: Fear of Childbirth and Postpartum Period Scale

**p < 0.01

## Discussion

We found a statistically significant relationship between the OCB-PPBC and the FCPPS scores. Eighteen per cent of the total variance in the obsessive and compulsive behaviors of the participants related to baby care in the postpartum period explained by the FCPPS scores (table 4) was one of the remarkable findings of this study. The reported literature has emphasized that the fear of childbirth and perinatal obsessive-compulsive disorder have negative effects on fetal, neonatal, and infant health. Besides, they negatively affect parenting, the functioning of relationships, and daily life in the postnatal period ^16^^-^^19^. Additionally, the obsessive and compulsive behaviors of mothers related to baby care may negatively affect their communication and attachment process with their infants ^18^.

The result of our study demonstrated that the fear of childbirth is a predictor of postpartum obsessive-compulsive disorder. The uncertainties of the COVID-19 pandemic have led women to be unable to go to health institutions, fear infections and think of the possibility of harming their baby or themselves in this process ^20^^,^^21^. Apparently, the COVID-19 pandemic has increased women’s fears of childbirth, as well as their obsessive and compulsive behaviors related to baby care.

The OCB-PPBC scores of this study increased significantly due to increased participant’s concerns about postpartum, breastfeeding, inadequate infant care, and infant and maternal health. Other related studies reported that the anxiety levels of new mothers regarding their babies are moderate to high ^22^^-^^24^. These results show that women are afraid to fail in postpartum baby care, and if their fears are not resolved, their obsessive and compulsive behaviors related to baby care will increase. These results also emphasize the importance of providing education starting from the antenatal period to develop baby care skills.

In this study, the mean of the FCPPS scores was 5.06 ± 1.46, fears of childbirth and the postpartum period were moderate in general. National and international studies have reported moderate or high childbirth anxiety levels in pregnant women ^3^^,^^22^^-^^26^. Many factors can affect childbirth and postpartum anxiety. In our study, we did not find a statistically significant relationship between the mean of the FCPPS scores and the participant’s age, education or employment status, economic status, family type, or chronic disease status. Unlike our results, some studies have demonstrated that age ^27^^,^^28^ and employment status ^22^ are related to the fear of childbirth. Different characteristics may be effective on childbirth and postpartum anxiety in cases of regional and cultural differences.

Among obstetric characteristics, parity is a well-known predictor of the fear of childbirth, where nulliparous women have more fear of childbirth than multiparous women ^22^^,^^25^^,^^26^. In line with this, one of the expected findings of this study was the significantly higher childbirth and postpartum anxiety levels of the primiparous participants than those of the multiparous (p < 0.05) (table 1). This result may be associated with the fact that women who became pregnant for the first time and are soon-to-be mothers have never experienced childbirth and baby care, and the process is uncertain for them.

Postpartum women are a riskier group regarding obsessive-compulsive disorder compared to the general population of women ^6^. The prevalence of obsessive-compulsive disorder in postpartum women without a previous diagnosis is 11.2%, and the rate of subclinical obsessive-compulsive disorder is 37.5% ^12^. Obsessive-compulsive disorder behaviors gradually increase during the pregnancy, childbirth, and postpartum periods, respectively. Several studies have provided evidence of the presence of obsessivecompulsive disorder symptoms in women in the perinatal period; however, studies involving a clear analysis of risk factors and causes are still lacking ^6^^,^^16^. The mean OCB-PPBC score of this study was 24.07 ± 8.70. The participants who were not working and those who were first-time mothers had high OCB-PPBC. The OCB-PPBC scores increased as the participants’ ages decreased. Evidence-based studies stated that women, especially those younger and experiencing motherhood for the first time were worried and concerned about the care of their babies and their self-efficacy and presented high obsessive-compulsive disorder rates ^17^^,^^29^^,^^30^.

In this study, the mean OCB-PPBC score of the participants who were not working was higher than the mean score of those who were working. Kabul and Çinar work conducted in the same province, provided similar results ^31^. In the qualitative study of Guy and Arthur (2020) on academic motherhood during the pandemic, one of the mothers stated that being at home for a long time increased her rate of obsessive-compulsive behaviors ^32^. Contrary to this result, in another research using the OCB-PPBC scale, the authors reported that the OCB-PPBC scores were significantly higher in employed mothers than in non-employed mothers ^33^. Studies examining this aspect are limited in number, so more studies focusing on employment status are needed.

The results of this study are based on the self-reports of the participants according to their responses to the FCPPS and the OCB-PPBC scales. We assumed the participants answered all questions and scales correctly. Another limitation was that the data were obtained at a single institution and collected during the COVID-19 pandemic which may have affected the results. Additionally, we did not distinguish between pregnant women with or without prior fertility problems. The last limitation of the study was the lack of assessment of the confounding factors affecting these two conditions. In line with these limitations, the results obtained in the study may apply only to this population. The third trimester of pregnancy is the time when the fear of childbirth and the postpartum period is intense, and the 4th-8th weeks of the postpartum period is when obsessive-compulsive disorder is severely experienced. Obtaining responses to the scales used in the study within the specified time frame constituted the strength of the study in terms of better describing the situation experienced by the mothers and reflecting the data accuracy more clearly.

Our results showed moderate levels of fear of childbirth and the postpartum period in the participants, as well as moderate levels of obsessive and compulsive behaviors regarding baby care. These behaviors increased as mother’s concerns about childbirth, their babies, and baby care during pregnancy increased. We observed that 18.0% of the total variance in the OCB-PPBC scores of the participants was explained by their FCPPS scores. The OCB-PPBC mean score of the unemployed participants was significantly higher, while both the FCPPS and the OCB-PPBC scores of the primiparous participants were higher and statistically significant. Based on these results, the reasons for the obsessive and compulsive behaviors of the participants were related to basic care concerns about childbirth, the baby, and baby care. In other words, pregnant women concerned about delivery and postpartum baby care are at risk of obsessive-compulsive behavior.

This finding pointed out the necessity of addressing the emotions and needs -experienced before and during childbirth- in the health evaluation of the mother and the baby after childbirth. It is recommended that all pregnant women be screened for fears of childbirth in the prenatal period. Identifying pregnant women with high fear levels as soon as possible and providing proactive education and support in the prenatal period is essential to promote maternal and infant health. Nurses should see the pregnancy period as an opportunity for mothers to prepare for baby care in the postpartum. They should prepare the mother for this period with proactive training.

The results of this study contribute with academic information to the fields of obstetrics, pediatrics, and psychiatric nursing by shedding light on maternal concerns about childbirth and obsessive-compulsive behaviors related to baby care during unstudied longitudinal periods between pregnancy, childbirth, and postpartum. It is important to study these concepts in terms of maternal and infant health. In this context, we think that these study results and recommendations will help to fill the gap on the subject. Considering the multidimensional nature of the fear of childbirth, all details and effects of it should be examined and evaluated in future studies. Additionally, identifying obsessive and compulsive behaviors related to baby care in the postpartum with observational and longitudinal studies will contribute to the clarification of the subject. Studies are also recommended to determine the potential longterm effects of anxiety about childbirth and the postpartum during pregnancy on the mother and the baby.
